# The Decrease of Leaf Dark Respiration during Water Stress Is Related to Leaf Non-Structural Carbohydrate Pool in *Vitis vinifera* L.

**DOI:** 10.3390/plants11010036

**Published:** 2021-12-23

**Authors:** Sergio Tombesi, Tommaso Frioni, Francesca Grisafi, Paolo Sabbatini, Stefano Poni, Alberto Palliotti

**Affiliations:** 1 Department of Sustainable Crop Production, Università Cattolica del Sacro Cuore, Via Emilia Parmense 84, 29122 Piacenza, Italy; tommaso.frioni@unicatt.it (T.F.); francesca.grisafi1@unicatt.it (F.G.); stefano.poni@unicatt.it (S.P.); 2 Department of Horticulture, Michigan State University, 1066 Bogue Street, East Lansing, MI 48824, USA; sabbatin@msu.edu; 3 Department of Agricultural, Food and Environmental Sciences, Università degli Studi di Perugia, Borgo 20 Giugno 74, 06154 Perugia, Italy; alberto.palliotti@unipg.it

**Keywords:** dark respiration, transpiration, water stress, plant physiology

## Abstract

Dark respiration (R_d_) is a fundamental plant process used to gain biomass and maintain plant physiological activity. It accounts for the metabolization of a large share of the carbon fixed by photosynthesis. However, R_d_ during conditions of severe plant water stress is still poorly understood. The decrease in leaf transpiration increases temperature, one of the most important drivers of leaf R_d_. On the other hand, water stress decreases the pool of leaf carbohydrates, which are the most important substrate for respiration. The aim of the present work was to determine the impact of water shortage on leaf R_d_ in grapevine and understand the driving factors in modulating leaf R_d_ response under plant water stress conditions. Water stressed vines had lower R_d_ as the water shortage severity increased. R_d_ was correlated with leaf temperature in well-watered vines. Instead, in water stressed vines, R_d_ correlated with leaf soluble sugars. The decrease of leaf R_d_ in water stressed vines was due to the decrease of leaf non-structural carbohydrate that, under water stress conditions, exerted a limiting effect on R_d_.

## 1. Introduction

DOY, day of the year; g_s_, stomatal conductance; Ψ_pd_, pre-dawn water potential; PPFD, photosynthetic photon flux density; P_n_, net photosynthesis; R_d_, dark respiration; RWC, relative water content; T_leaf_, leaf temperature; WS, water stress; WW, well-watered.

Mitochondrial respiration (also referred to as dark respiration) is the controlled oxidation of reduced carbohydrates, producing CO_2_, reducing equivalents (NAD(P)H and FADH2) and resulting in respiratory O_2_ consumption and ATP production [1]. In woody plants, between 25% and 75% of the CO_2_ fixed during photosynthesis can be released back to the atmosphere by plant respiration each year [2,3,4,5]. Respiration is utilized by plants to gain biomass (growth respiration) and to provide energy for all plant physiological processes that do not result in a net increase in plant dry matter, such as maintenance of ion gradients across membranes and the resynthesis of degraded organic compounds [6]. Plant tissues have a specific respiration rate and generally the highest is measured in the leaves, and it accounts for half of the plant respiration [2].

The amount of CO_2_ that is lost daily through dark respiration (R_d_) is related to environmental conditions [7] and in particular to temperature regimes [4,8]. The increase of temperature due to global warming can have a substantial impact on plant respiration [9,10] and this may have consequences on plant ability to fix CO_2_ [11] and, ultimately, on agricultural crop productivity. Further, global warming is increasing daily mean temperature but also the frequency of extreme heat events. Furthermore, rainfall patterns are becoming more erratic exposing plants to recurrent drought stress that often reduce plant yield [11]. Drought stress and heat waves negatively impact several processes; plant growth is reduced during the early stages of water shortage [12], transpiration and photosynthesis is limited [13] and plant carbon partitioning to different organs is altered [14]. Water stress affects leaf R_d_ too. Although the respiration rate is mainly limited by extreme temperatures, an important role is also played by several other factors; e.g., substrate availability, rate of the respiration processes, use of the respiratory products and the efficiency of the respiratory pathway [2,15]. Water stress increases leaf temperature due to a reduced leaf transpiration [16] and reduces the respiration substrate pool, due to a reduced photosynthetic assimilation [17]. This can cause R_d_ to be limited by different factors according to the level of water availability.

In literature, there is poor consensus on the effect of water shortage on plant respiration [18]. Under water stress, respiration was reported to decrease [19,20,21,22,23,24,25], or to be almost unaffected [26,27] or even to increase [28,29,30]. Moreover, a biphasic response of respiration in plants with different water content was proposed: R_d_ decreases when the water stress is mild (soil RWC < 50%) and it increases when the water stress was more severe [18].

In grapevine, several studies reported that R_d_ decreased as water stress rose [3,31,32,33]. However, Perez-Martin et al. [34] observed a consistent increase in respiration due to water shortage stress and Gomez-del-Campo et al. [14] reported an initial increase in night respiration and subsequent decrease as the water stress became more severe. Indeed, considering that water stress causes consistent variation in several factors affecting R_d_, such as leaf temperature and carbohydrate availability [35], our hypothesis was that dark respiration variation during water stress can be caused by the indirect effect of water stress on leaf temperature and leaf carbohydrate status rather than to the water stress itself.

The aim of the present work was to determine the effect of soil water deprivation on leaf R_d_. We focused our research on determining the role of leaf temperature and leaf carbohydrate content in R_d_ regulation during water stress in grapevines.

## 2. Results

### 2.1. Water Deprivation and Gas Exchange over the Experiment

**Figure 1:**
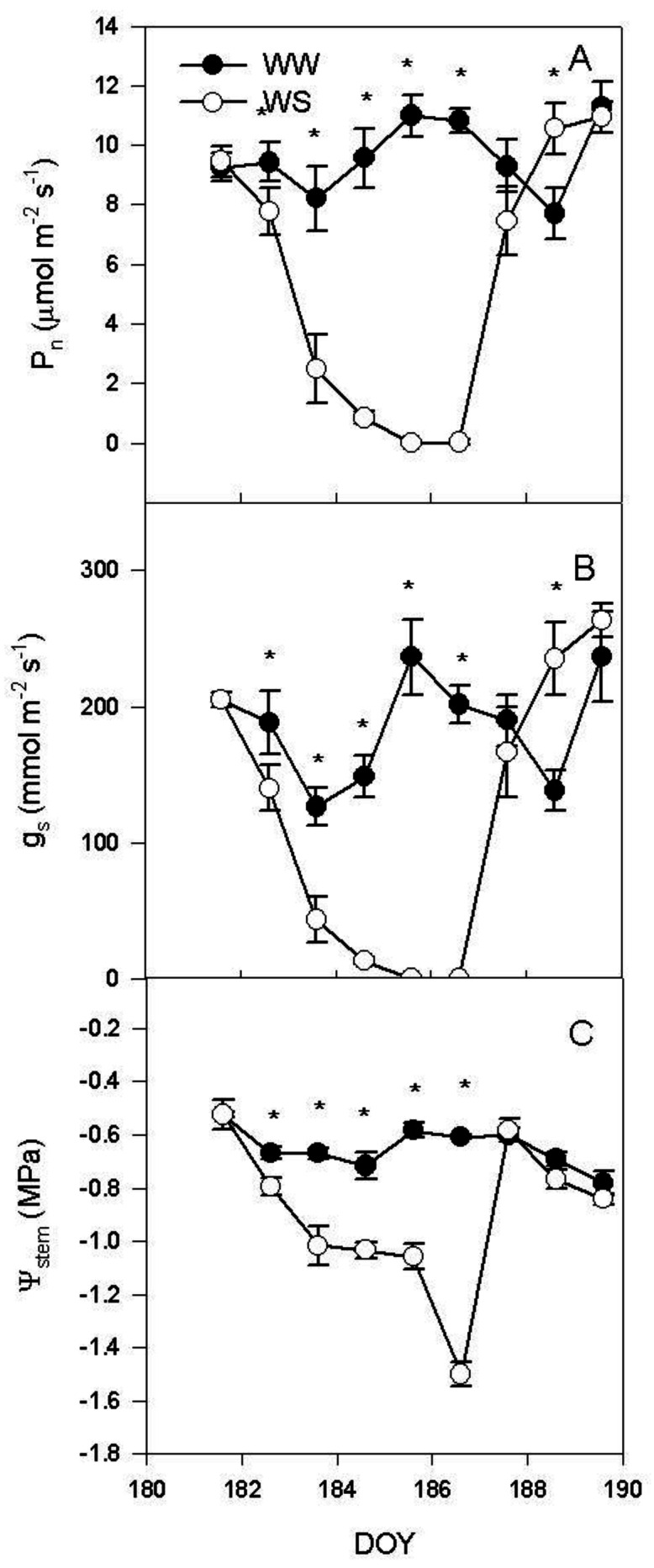
(**A**) net photosynthesis (P_n_), (**B**) stomatal conductance (g_s_), and (**C**) stem water potential at midday (Ψ_stem_) recorded on mature leaves of well-watered (WW) and water stress vines (WS) during the experiment. Each point is the mean of six vines ±SE. Points marked by asterisk are different per *p* < 0.05 (*t*-test).

Net photosynthesis (P_n_) and stomatal conductance (g_s_) decreased during water stress from DOY 182 to DOY 186 (Figure 1A,B).

Upon re-watering (DOY 187), P_n_ and g_s_ were similar between WS and WW vines except at DOY 188 when the WS treatment had higher P_n_ and g_s_ than the WW vines. Ψ_stem_ in WS vines decreased during water stress up to −1.5 ± 0.04 MPa and reached values like the WW treatment soon after re-watering (Figure 1C). In WW vines, Ψ_stem_ ranged between −0.52 ± 0.01 and −0.78 ± 0.06 MPa.

**Figure 2:**
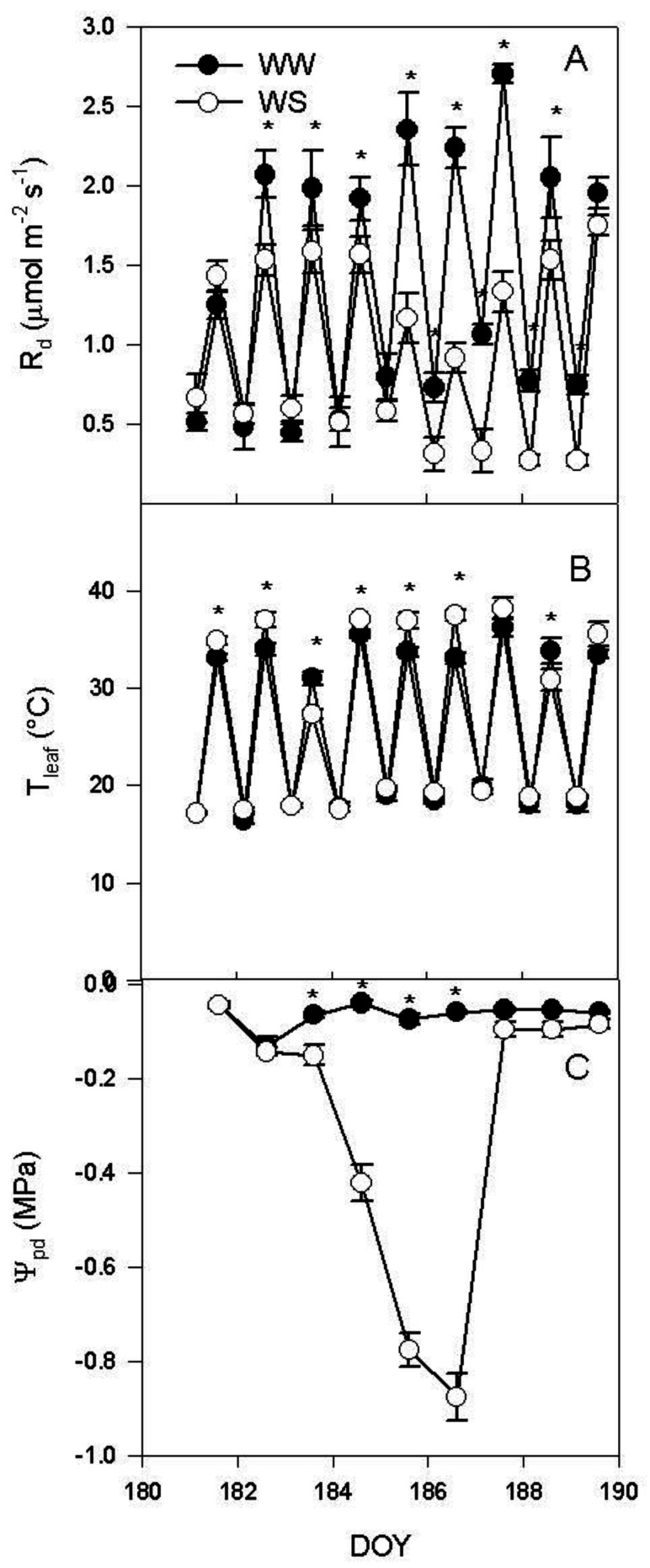
(**A**) Dark respiration (R_d_), (**B**) leaf temperature (T_leaf_) at 4:00 a.m. and midday, and (**C**) pre-dawn water potential (Ψ_pd_) recorded on mature leaves of well-watered (WW) and water stressed vines (WS) during the experiment. Each point is the mean of six vines ± SE. Points marked by asterisk are different per *p* < 0.05 (*t*-test).

At midday, leaf dark respiration (R_d_) was significantly lower in WS vines than in WW vines starting from DOY 182 until DOY 188 (Figure 2A). R_d_ at 4:00 a.m. was significantly lower in WS leaves when compared with WW vines between DOY 186 and DOY 189. At midday, leaf temperature (T_leaf_) was higher in WS than in WW on DOY 181 and 182 and between DOY 185 and DOY 186 (Figure 2B).

On DOY 183 and 188, T_leaf_ was higher in WW leaves in comparison with WS leaves. At 4:00 a.m., T_leaf_ was similar in WW and WS leaves during the entire experimental period. Water potential at pre-dawn (Ψ_pd_) was higher in WW than in WS between DOY 183 and 186 (Figure 2C). In WS, Ψ_pd_ ranged between −0.04 ± 0.001 MPa and −0.87 ± 0.05 MPa.

**Figure 3:**
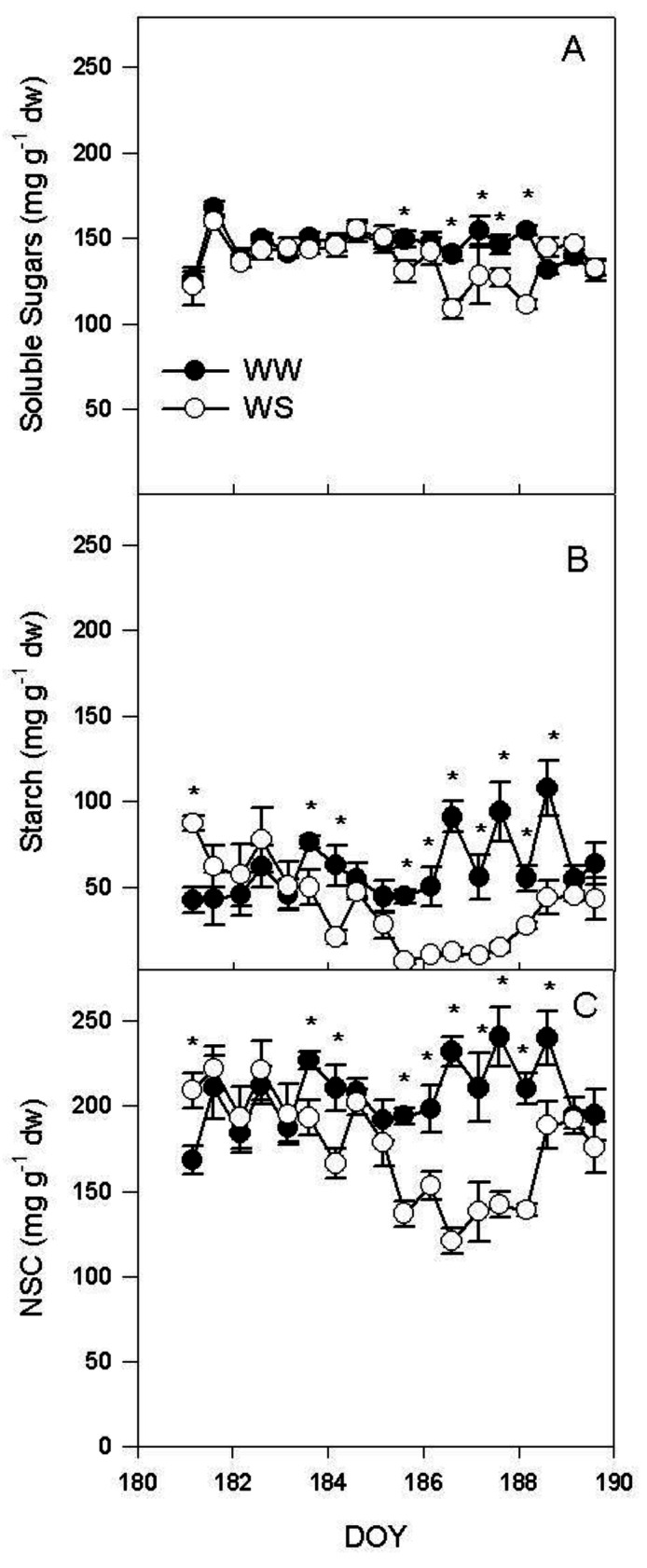
(**A**) soluble sugars, (**B**) starch and (**C**) non-structural carbohydrates (NSC) measured on mature leaves of well-watered (WW) and water stressed vines (WS) during the experiment. Each point is the mean of six vines ±SE. Points marked by asterisk are different per *p* < 0.05 (*t*-test).

In leaves, soluble sugars were lower in WS than in WW at midday on DOY 185 and between DOY 186 and DOY 187 (Figure 3A).

Starch was lower in WS than in WW treatment between midday on DOY 183 and at 4:00 a.m. on DOY 184 and between midday on DOY 185 and midday in DOY 188 (Figure 3B). Non-structural carbohydrates (NSC) were lower in WS than in WW treatments between midday at DOY 183 and at 4:00 a.m. at DOY 184 and between midday on DOY 185 and midday on DOY 188 (Figure 3C).

### 2.2. R_d_ Regression with T_leaf_ in WW Vines

**Figure 4:**
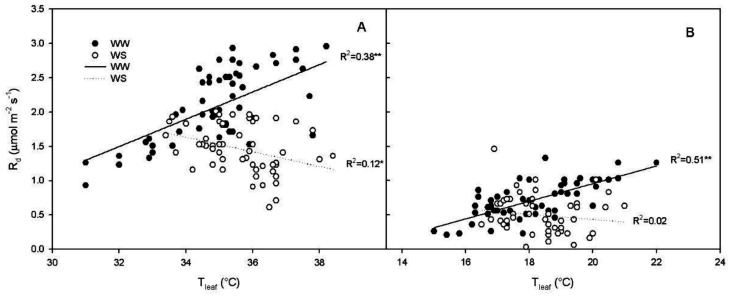
Relationship between leaf dark respiration (R_d_) and leaf temperature (T_leaf_) in well-watered (WW) and water stressed (WS) vines at (**A**) midday (y = −4.87 + 0.20x, R^2^ = 0.38 *p* < 0.0001; y = 5.26−0.10x, R^2^ = 0.12 *p* = 0.01, respectively) and (**B**) at 4:00 a.m. (y = −1.61 + 0.12x, R^2^ = 0.51 *p* < 0.0001; y = 1.20−0.04x, R^2^ = 0.02 *p* = 0.29, respectively). Regressions not statistically significant are represented as dot-lines. * indicates *p* < 0.05; ** indicates *p* < 0.0001.

There was a positive regression between R_d_ and T_leaf_ at midday in WW vines (R^2^ = 0.38, *p* < 0.001), while in WS vines the regression, thought significant, was negative (R^2^ = 0.12, *p* < 0.01) (Figure 4A).

At 4:00 a.m., there was a linear, positive regression between R_d_ and T_leaf_ in WW vines (R^2^ = 0.51, *p* < 0.001), while in WS vines the regression was not significant (R^2^ = 0.02, *p* = 0.29) (Figure 4B).

### 2.3. Rd Regression with Soluble Sugars in WS Vines

**Figure 5:**
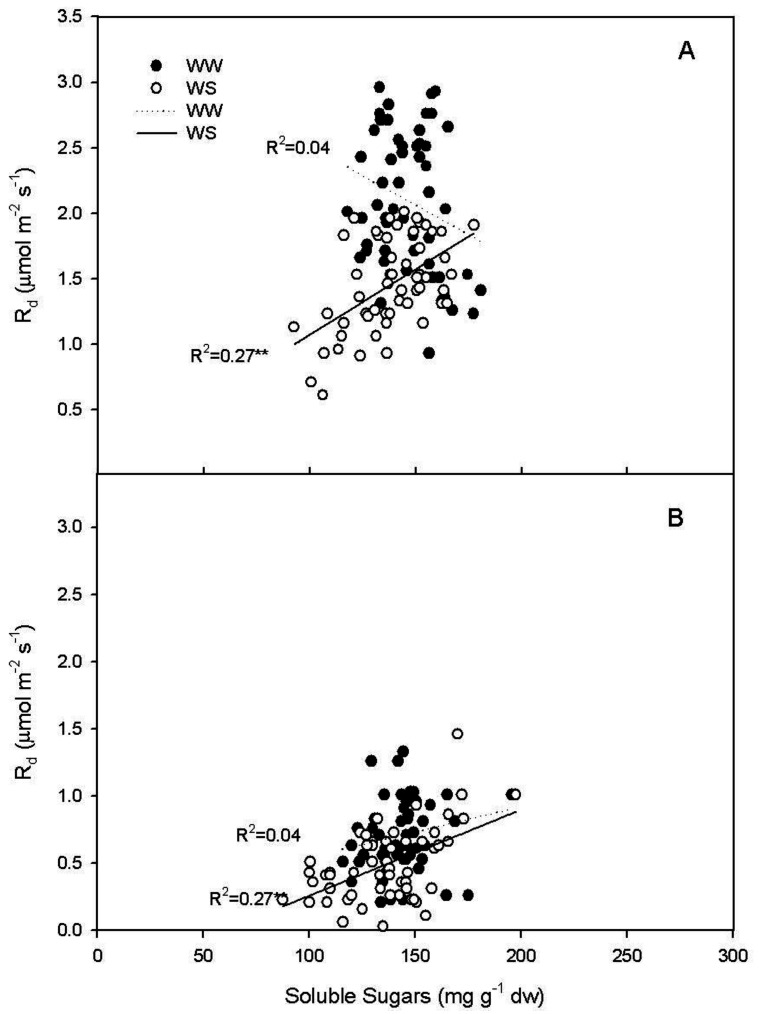
Relationship between leaf dark respiration (R_d_) and leaf soluble sugars in well-watered (WW) and water stressed (WS) vines at (**A**) midday (y = 3.40−8.87x, R^2^ = 0.06 *p* = 0.07; y = 0.07 + 10.01x, R^2^ = 0.27 *p* < 0.0001, respectively) and (**B**) at 4:00 a.m. (y = 0.17 + 3.75x, R^2^ = 0.04 *p* = 0.15; y = −0.37 + 6.39x, R^2^ = 0.27 *p* < 0.0001, respectively). * indicates *p* < 0.05; ** indicates *p* < 0.0001.

A positive regression was also reported between R_d_ and leaf soluble sugars at midday in WS vines (R^2^ = 0.27, *p* < 0.001), while in WW vines the regression was not significant (R^2^ = 0.06, *p* = 0.07) (Figure 5A).

At 4:00 a.m., there was a linear, positive regression between R_d_ and leaf soluble sugars in the WS treatment (R^2^ = 0.27, *p* < 0.001), while in WW treatment the regression was not significant (R^2^ = 0.04, *p* = 0.15) (Figure 5B).

### 2.4. Relationship between Rd, Ψ_pd_ and Non-Structural Carbohydrates

**Figure 6:**
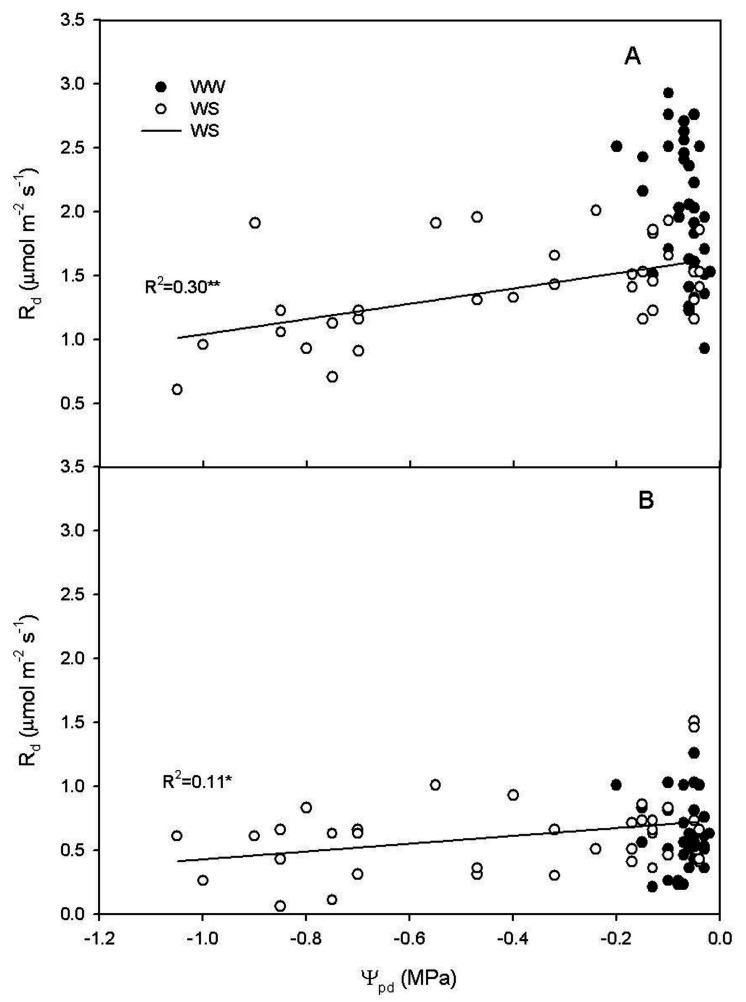
Relationship between leaf dark respiration (R_d_) and pre-dawn water potential (Ψ_pd_) in water stressed (WS) vines at (**A**) midday (y = 1.64 + 0.59x, R^2^ = 0.30 *p* < 0.0001) and (**B**) at 4:00 a.m. (y = 0.73 + 0.31x, R^2^ = 0.11 *p* = 0.04) during water stress from DOY 181 to DOY 187. * indicates *p* < 0.05; ** indicates *p* < 0.0001.

In WS vines, a relationship between R_d_ at midday and Ψ_pd_ (R^2^ = 0.30, *p* < 0.001) was evidenced by a linear regression (Figure 6A) and, though barely significant, the linear regression was reported also with data recorded at 4:00 a.m. (R^2^ = 0.11, *p* = 0.04) (Figure 6B).

**Figure 7:**
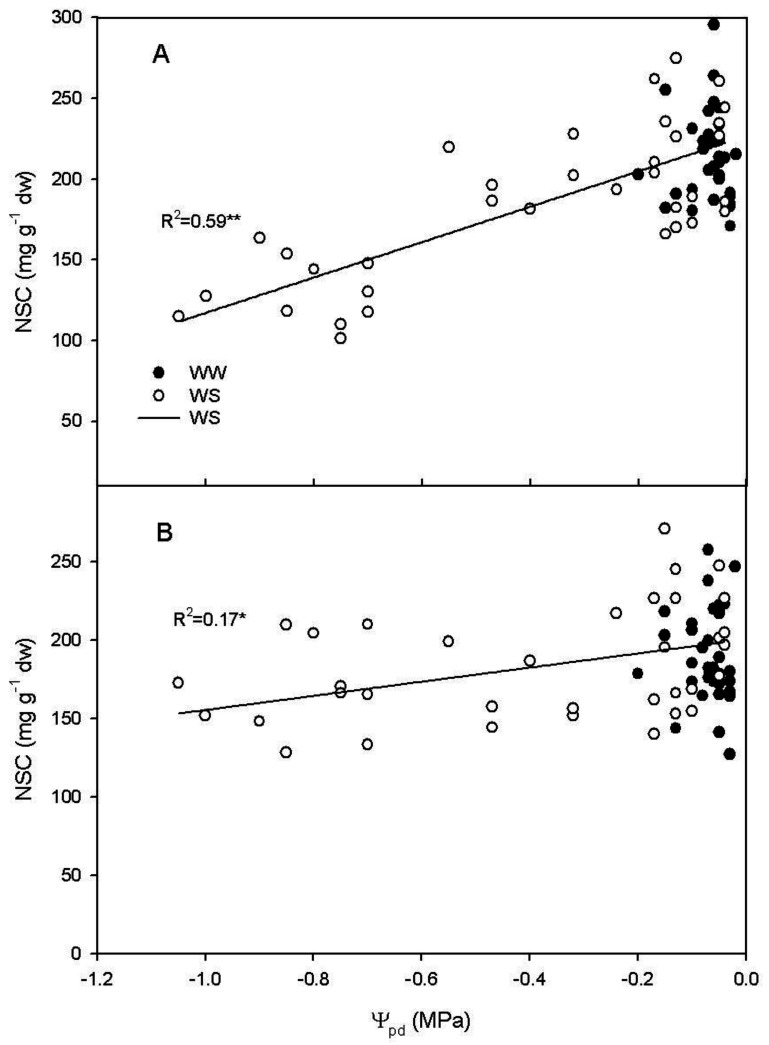
Relationship between non-structural carbohydrates (NSC) and pre-dawn water potential (Ψ_pd_) in water stressed (WS) vines at (**A**) midday (y = 0.23 + 0.11x, R^2^ = 0.59 *p* < 0.0001) and at (**B**) 4:00 a.m. (y = 0.20 + 0.04x, R^2^ = 0.17 *p* = 0.011) during water stress from DOY 181 to DOY 187. * indicates *p* < 0.05; ** indicates *p* < 0.0001.

In the WS treatment, a relation between leaf NSC at midday and Ψ_pd_ was described by a linear regression (R^2^ = 0.59, *p* < 0.001) (Figure 7A), and reported between leaf NSC at 4:00 a.m. and Ψ_pd_ (R^2^ = 0.17, *p* = 0.011) (Figure 7B).

## 3. Discussion

The withholding of the irrigation induced a rapid decrease in Ψ_stem_ that caused a complete stomata closure and a consequent severe reduction of the photosynthetic activity on DOY 185 (Figure 1). During the water stress, R_d_ was particularly reduced at midday (Figure 2A). The lower R_d_ measured at midday was inconsistent with the higher temperature recorded in WS leaves when compared to the WW ones (Figure 2A,B). In the condition of reduced water availability, as indicated by the Ψ_pd_ (Figure 2C), there was a limited leaf transpiration and thermoregulation capacity. WS vines had lower soluble sugar content than WW vines (Figure 3A). Instead, the effect of the water stress was more evident on starch content, which decreased during the early stages of the water stress, mirroring the dynamic of leaf photosynthesis. Overall, NSC decreased as consequence of water stress.

The major environmental driver of R_d_ is temperature [8]. In our experiment this was true in well-watered plants, and, in fact, R_d_ was positively correlated with leaf temperature (T_leaf_) when measured at midday and at 4: 00 a.m. (Figure 4). Contrarily, in WS vines there was no positive regression (Figure 4) between R_d_ and T_leaf_, indicating that, under WS condition, leaf temperature was not the limiting factor driving R_d_. This does not mean that temperature is not influencing Rd, but that under WS the influence of temperature is less limiting than that of other factors and for this reason it explains a minimal percentage of the variability of the experimental sample. These results are to some extent in disagreement with those of Escalona et al. [3] which reported similar respiratory quotient in well-watered and leaves of vines subject to mild water stress (WS was set in order to keep gs > 75 mmol m^−2^ s^−^^1^). In our experiment, the WS treatment reached complete stomatal closure and R_d_ was significantly correlated to leaf soluble sugars. However, such regression was not significant in the leaves of the WW treatment (Figure 5). This result suggests that under water stress the limiting factor for R_d_ is represented by the amount of total carbohydrates available as substrate for respiration. Contrarily, under WW conditions leaf temperature plays a more significant role in limiting respiration. This accounts also for the slower recovery of R_d_ after re-watering when compared with the recovery of P_n_ and g_s_ (Figure 1 and Figure 2). The slow recovery of R_d_ was like the dynamic of the recovery of soluble sugar content that slightly preceded that of starch in the leaf. The decrease of NSC in leaves during water stress agrees with what was previously reported in grapevine [36], but it is in contrast with what has been reported in *Eucalyptus saligna*, in which starch content increased during the early stages of water stress [37].

In our experiment, R_d_ at midday significantly decreased as the Ψ_pd_ became more negative, while no regression was evidenced at 4:00 a.m. (Figure 6). The same trend was observed for NSC; at midday, NSC significantly decreased as the Ψ_pd_ became more negative, but, at 4:00 a.m., NSC slightly decreased during the water stress period (Figure 7). This indicates that R_d_ progressively decreased as the water availability decreased, in accordance with previous works [3,31,32,33]. Although, in such studies this relationship was not fully explained [14,31,32,33]. Escalona et al. [3] speculated that R_d_ reduction in roots and stems was linked to a reduced Q_10_ related to the decrease of carbohydrate export from the leaves. Our experiment demonstrated that the reduction of leaf R_d_ over Ψ_pd_ was related to the dynamic of NSC content in the leaf, that decreased over the water stress period. The decrease of NSC during the water stress was more consistent at midday than at 4:00 a.m. and this explains the weaker decrease of R_d_ during water stress recorded at 4:00 a.m. In particular, the natural daily variation in NSC and the effect of water stress in reducing the NSC daily variation due to the reduced photosynthetic activity, could explain the contradictory results reported in literature. Indeed, respiration was reported to decrease [19,20,21,22,23,24,25] or to be almost unaffected [26,27] or even to increase [28,29,30] under water stress. Our results indicates that the reduction of leaf R_d_ occurs when the water stress induces a significant reduction of NSC, limiting R_d_. If NSC are not reduced, the increase of leaf temperature induced by the reduction in leaf transpiration can cause an increase of R_d_, juxtaposing the indirect effect of water stress on R_d_. Furthermore, these data suggest that the combination of different time of measurement (dawn vs. midday) with water stress severity and the daily dynamic of leaf carbohydrate accumulation, area all influencing the leaf R_d_.

## 4. Materials and Methods

### 4.1. Plant Material

The experiment was carried out in 2015 in an outdoor area of the Department of Agricultural, Food and Environmental Sciences, University of Perugia, located in the urban area of the city of Perugia, central Italy (43° 10′ 30″ N 12° 39′ 45″ E, 405 m a.s.l). Twelve 60 L pots were filled with loamy soil having a field capacity of 30.2% {(volume water/volume soil) × 100} and a wilting point of 16.7%. Each pot contained a 5-year-old vine of cv. Sangiovese (clone VCR30, grafted on 1103 Paulsen). Vines were winter spur-pruned leaving three spurs per vine carrying two buds each. During vine growth, developing shoots were directed upright using suitable stakes. Pots were maintained at field capacity throughout the experiment by an automated water-supply system providing water to each pot for 1 min three times per day (08:00 h, 13:00 h and 18:00 h, 6 L per vine per day).

On six vines (WS), irrigation was withheld on 31 June (DOY 182) to 5 July (DOY 186). On 6 July (DOY 187) irrigation was resumed. The remaining vines were kept well irrigated throughout the whole experiment (WW).

### 4.2. Gas Exchange

Stomatal conductance (g_s_) and net photosynthesis (P_n_) measurements were carried out on fully expanded fully mature leaves grown between the 4th and the 10th node. Measurements were performed between 12:00 a.m. and 1:00 p.m., from 30 June until 8 July on one representative leaf per vine on six vines per treatment (*n* = 6) using an open gas exchange system (ADC-System, LCA-3, Hoddesdon, UK) equipped with a Parkinson leaf chamber (window size of 11.2 cm^2^). Measurements were conducted under saturating sun light conditions (PPFD > 1200 µmol photons m^−2^ s^−1^). Dark respiration (R_d_) was measured at 4:00 a.m. at night (complete darkness, PPFD = 0 μmolm^−2^·s^−1^) and at midday at ambient [CO_2_]. At midday leaves were covered by an aluminium foil 15 min before enclosure in the leaf chamber. Leaves were then sampled for non-structural carbohydrate determination.

### 4.3. Water Potential Measurements

On the same leaves used for gas exchange, leaf water potential was measured by a pressure chamber (Soilmoisture Corp, Santa Barbara, CA, USA). Stem water potential (Ψ_stem_) was measured over the same days and daytime of gas exchange on each vine on one mature leaf that had been wrapped in plastic film and aluminium foil 2 h prior to the measurements. Water potential values measured at 4:00 a.m. are reported as pre-dawn water potential (Ψ_pd_).

### 4.4. Non-Structural Carbohydrate Determination

Leaves used for gas exchange (n = 6) were immediately placed in liquid nitrogen and then stored in a freezer at −80 C°. Then, the material was weighted and lyophilized (LIO5P, 5Pascal, Trezzano, Italy). Lyophilized material was weighted (dry weight) and grinded (MF10, IKAlabortechnik, Staufen, Germany). 0.01 g of leaf powder were placed in 15 mL tubes and added with a solution of ethanol 80% and placed in a warm bath with temperature set at 80 C° for 1 h. After 10 min of centrifugation at 10,000 rpm, 10 μL of supernatant was sampled and used for the determination of alcohol soluble sugars by the anthrone method [38,39]. For starch determination, pellet material was then washed with sodium acetate buffer and then added with 0.5 mL of sodium acetate buffer. Tubes were placed in warm bath with temperature set at 80 C° for 1 h. One millilitre of solution of amyloglycosidase and α-amylase in 0.05 M sodium acetate buffer was added as described by Chow and Landhäusser (2004) [40] and bath temperature was set at 50 C°. Soluble sugar content was then measured on the supernatant by the anthrone method as previously described.

### 4.5. Statistical Analysis

Data were analysed by linear and non-linear regression analysis using Sigmaplot 8.0 (SystatSoftware Inc., San Jose, CA, USA), and one way ANOVA was performed on dependent and independent variables in order to test the regression significance. Treatments were analysed by one-way ANOVA with significance level set at 0.05 and means were separated by Tukey’s w-procedure at *p* = 0.05.

## 5. Conclusions

Water deprivation caused a decrease in grapevine transpiration and a consequent increase in leaf temperature and a consequent decrease in leaf dark respiration. Such reduction was related to the reduction of carbohydrates caused by the reduction of photosynthetic activity. Under water stress condition, the reduced availability of carbohydrates appears to play a larger role than temperature in limiting leaf dark respiration of grapevines.

## Data Availability

Data available on request.
